# P-67. Bacteremia in the Intensive Care Unit: Pathogen Profiles and Antimicrobial Resistance Trends from January 2022 to June 2024

**DOI:** 10.1093/ofid/ofaf695.296

**Published:** 2026-01-11

**Authors:** Nawaf Abdulla, Meghana Sarikonda, Sruthi Menon, Murali Alagesan, Ajay Anthur Nair, Aruloli Mohambourame

**Affiliations:** PSG Institute of Medical Sciences and Research, Coimbatore, Tamil Nadu, India; PSG Institute of Medical Sciences and Research, Coimbatore, Tamil Nadu, India; PSG IMSR and Hospitals, Coimbatore, Tamil Nadu, India; PSG Institute of Medical Sciences and Research, Coimbatore, Tamil Nadu, India; PSG IMSR and Hospitals, Coimbatore, Tamil Nadu, India; PSG Institute of Medical Sciences and Research, Coimbatore, Tamil Nadu, India

## Abstract

**Background:**

Bacteremia is a major concern in ICUs due to high morbidity and mortality, particularly with multidrug-resistant organisms. Rising antimicrobial resistance further complicates management, limiting therapeutic options, prolonging ICU stays, increasing costs, and elevating mortality rates. Monitoring pathogen profiles and resistance trends is crucial for optimising antibiotic stewardship and guiding empirical therapy. This study analyses resistance patterns and pathogen prevalence in bacteremia in an ICU setting in India over a 30-month period.

**Figure:**
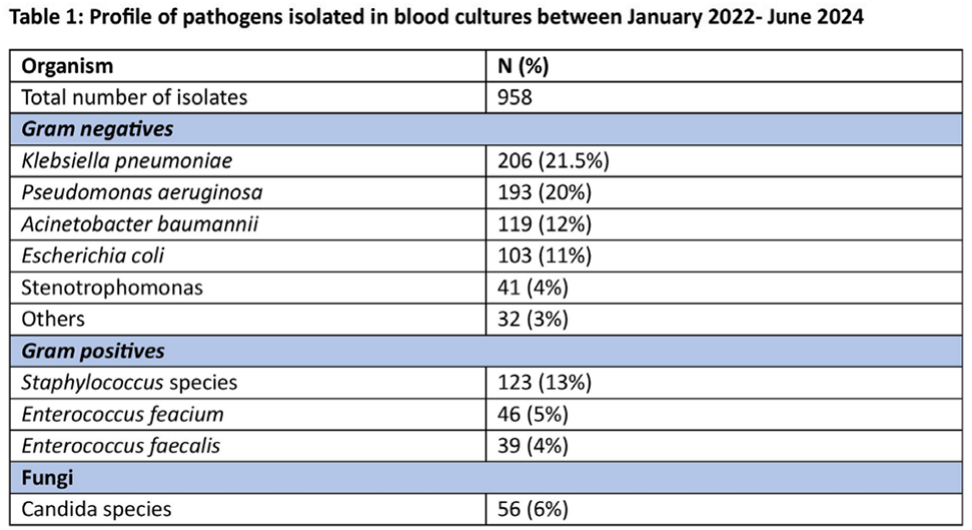

**Figure:**
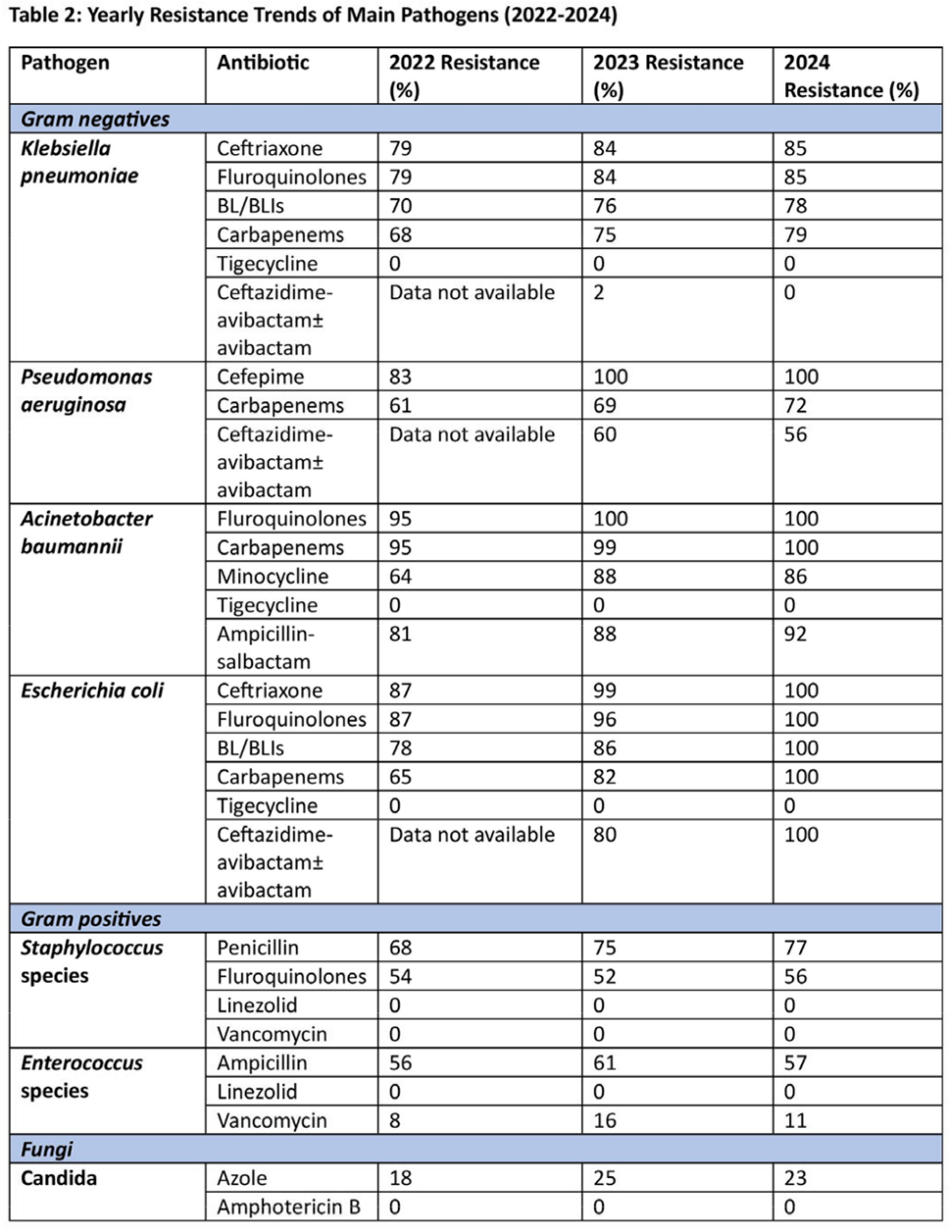

**Methods:**

A retrospective analysis was conducted in a tertiary care ICU in India. Blood culture reports of ICU patients (Jan 2022–June 2024) were collected. Pathogen identification and antimicrobial susceptibility testing followed standard microbiological procedures.

**Results:**

Among 958 blood isolates, Gram-negative bacteria constituted 71.5%, with Klebsiella pneumoniae (21.5%), Pseudomonas aeruginosa (20%), Acinetobacter baumannii (12%), and Escherichia coli (11%) as the predominant pathogens. Gram-positive bacteria made up 22%, mainly Staphylococcus spp. (13%) and Enterococcus faecium (5%). Fungal infections, primarily Candida spp., accounted for 6%.

Antimicrobial resistance among Gram-negative bacteria was notably high and increasing. Carbapenem resistance reached 79% in Klebsiella pneumoniae, 72% in Pseudomonas aeruginosa, and 100% in Acinetobacter baumannii and Escherichia coli by 2024. Other antibiotic classes, including fluoroquinolones and beta-lactam/beta-lactamase inhibitors, also showed increasing resistance over time, limiting treatment options. Gram-positive bacteria retained susceptibility to linezolid and vancomycin.

**Conclusion:**

The study highlights a concerning rise in antimicrobial resistance, particularly among Gram-negative organisms in an Indian ICU, complicating treatment strategies. The escalating resistance rates, especially to carbapenems, call for exploration of alternative therapeutic options and the development of novel antimicrobials to improve patient outcomes in critical care settings. The findings underscore the urgent need for targeted antimicrobial stewardship and stringent infection control measures.

**Disclosures:**

All Authors: No reported disclosures

